# TRP-2 / gp100 DNA vaccine and PD-1 checkpoint blockade combination for the treatment of intracranial tumors

**DOI:** 10.1007/s00262-024-03770-x

**Published:** 2024-07-02

**Authors:** Joshua R. D. Pearson, Carles Puig-Saenz, Jubini E. Thomas, Lydia D. Hardowar, Murrium Ahmad, Louise C. Wainwright, Adam M. McVicar, Victoria A. Brentville, Chris J. Tinsley, A. Graham Pockley, Lindy G. Durrant, Stephanie E. B. McArdle

**Affiliations:** 1 John Van Geest Cancer Research Centre, Nottingham Trent University, Nottingham, UK; 2 Department of Biosciences, School of Science and Technology, Nottingham Trent University, Nottingham, UK; 3 Bioscience Support Facility, School of Science and Technology, Nottingham Trent University, Nottingham, UK; 4 Scancell Ltd, Unit 202, Bellhouse Building, Oxford Science Park, Sanders Road, Oxford, OX4 4GA UK

**Keywords:** Brain tumor, Cancer vaccine, Glioblastoma multiforme, Immune checkpoint blockade, PD-1, TRP-2

## Abstract

Intracranial tumors present a significant therapeutic challenge due to their physiological location. Immunotherapy presents an attractive method for targeting these intracranial tumors due to relatively low toxicity and tumor specificity. Here we show that SCIB1, a TRP-2 and gp100 directed ImmunoBody® DNA vaccine, generates a strong TRP-2 specific immune response, as demonstrated by the high number of TRP2-specific IFNγ spots produced and the detection of a significant number of pentamer positive T cells in the spleen of vaccinated mice. Furthermore, vaccine-induced T cells were able to recognize and kill B16^HHDII/DR1^ cells after a short *in vitro *culture. Having found that glioblastoma multiforme (GBM) expresses significant levels of *PD-L1* and *IDO1*, with *PD-L1* correlating with poorer survival in patients with the mesenchymal subtype of GBM, we decided to combine SCIB1 ImmunoBody® with PD-1 immune checkpoint blockade to treat mice harboring intracranial tumors expressing TRP-2 and gp100. Time-to-death was significantly prolonged, and this correlated with increased CD4^+^ and CD8^+^ T cell infiltration in the tissue microenvironment (TME). However, in addition to PD-L1 and IDO, the GBM TME was found to contain a significant number of immunoregulatory T (Treg) cell-associated transcripts, and the presence of such cells is likely to significantly affect clinical outcome unless also tackled.

**Supplementary Information:**

The online version contains supplementary material available at 10.1007/s00262-024-03770-x.

## Introduction

Intracranial tumors present a unique challenge due to their physiological location and the sensitivity of the brain as an organ. For therapy to be beneficial to patients harboring intracranial tumors, the therapy needs to penetrate the brain parenchyma and cause little to no damage to the brain itself. Many chemical therapies fail due to their inability to cross the blood brain barrier and the potential deleterious effects they have on the normal surrounding brain tissue. Immunotherapy presents an attractive therapeutic modality due to its tumor specificity and the ability of activated immune cells to cross the blood brain barrier [1]. Glioblastoma multiforme (GBM) is the most frequently occurring primary brain tumor and the prognosis for patients is poor with the median survival after diagnosis being 12–15 months [2, 3]. Many other types of tumors can also metastasize into the brain. Much like primary brain tumors, brain metastases carry an equally dismal prognosis [4–6]. Both brain tumors and brain metastases are treated in a similar manner, with surgical removal of the tumor being performed (where possible) followed by radiotherapy and, in some cases, chemotherapy

SCIB1 ImmunoBody® (Scancell Limited, Nottingham, UK) is a DNA vaccine encoding a human IgG1 antibody with three epitopes from gp100 and one from TRP-2 engineered into its complementarity determining regions (CDR). There are two HLA*0201 epitopes, one from TRP-2_180–188_ (SVYDFFVWL) which also is H-2 Kb restricted and one from gp100_178-186_ (MLGTHTMEV), and two CD4 epitopes, an HLA-DR4 restricted gp100_44-59_ epitope (WNRQLYPEWTEAQRLD) and a gp100_173–190_ epitope (GTGRAMLGTHTMEVTVYH) restricted by HLA-DR7, HLA-DR53 and HLA-DQ6 [7, 8]. ImmunoBody® induces a stronger immune response than equivalent peptide vaccines, whole antigen DNA vaccines and peptide-pulsed dendritic cells (DCs) [7, 9, 10]. The IgG1 molecule encoded by SCIB1 acts as a carrier protein for the immunogenic TRP-2 and gp100 peptides, which are cross presented to CD4^+^ and CD8^+^ T cells after uptake into and processing by DCs [9]. Based on promising results from pre-clinical melanoma models, a phase I/II trial of the SCIB1 ImmunoBody® in patients with melanoma demonstrated peptide-specific T cell responses in 88% of treated patients and that SCIB1 therapy was well tolerated [11]. TRP-2 and gp100 antigens represent excellent targets as their expression is not exclusive to melanoma, with expression also being seen in GBM [12–14]. This offers the possibility that the SCIB1 ImmunoBody® is effective in patients whose GBM tumors express TRP-2 and/or gp100.

The binding of programmed death protein 1 (PD-1) expressed on T cells to its cognate ligands programmed death ligands 1 and 2 (PD-L1 and PD-L2) on tumors abrogates the immune response—so called ‘checkpoint inhibition’. The blockade of this interaction with monoclonal antibodies has been approved for use in many cancer types and is also being investigated as part of combinatorial therapy in numerous cancer settings. The combination of SCIB1 with PD-1 blockade has been shown to result in a strong anti-tumor effect against murine B16-F1 melanoma cells expressing human HLA-DR4 implanted subcutaneously in transgenic mice [8]. Although the efficacy of PD-1/PD-L1 blockade as monotherapy for intracranial tumors has to date been limited [15–17], it presents an attractive adjunct for active immunotherapy. For example, PD-1 blockade improves responses to active immunotherapy in an orthotopic murine GBM model [18].

This study examined the immunogenicity of SCIB1 ImmunoBody® in a humanized C57BL/6 HHDII/DR1 mouse model and its ability to generate T cells having the capacity to reject/delay the growth of established intracranial tumors in vivo when combined with PD-1 immune checkpoint blockade.

## Materials and methods

### Cells and cell culture

The B16^HHDII/DR1^ cell line was a generous gift from Scancell Ltd (Nottingham, UK). B16^HHDII/DR1^ cells are derived from B16-F1 murine melanoma cells that have been genetically modified so that they no longer express murine MHC. Murine MHC was knocked out using zinc-finger nuclease technology and cells were stably transfected with plasmids encoding human HHDII and DR1 molecules. These cells were cultured in RPMI-1640 (Lonza, cat#: BE12-167F) supplemented with 10% v/v FBS (Gibco, cat#: A5670701), 2 mM L-glutamine (Lonza, cat#: BE17-606E), 300 µg/mL Hygromycin B (ChemCruz, cat#: sc-29067) and 500 µg/mL Geneticin (G418) Sulfate (ChemCruz, cat#: sc-29065). B16^HHDII/DR1^ cells were stably transfected with the *Luciferase* gene and transfected cells selected for using 550 µg/mL Zeocin (InvivoGen, cat#: ant-zn-1).

Splenocytes were cultured in T cell medium – RPMI-1640 supplemented with 10% v/v FBS, 2 mM L-glutamine, 20 mM HEPES buffer (Sigma, cat#: 83,264), 100 units/mL penicillin + 100 µg/mL streptomycin (Lonza, cat# DE17-602E) and amphotericin B (Lonza, cat#: 17-836R).

The SF-188 and SEBTA-027 human GBM cell lines were a generous gift from the University of Portsmouth Neuro-Oncology Group (Emeritus Professor Geoffrey Pilkington). SF-188 was derived from an 8-year-old male with stage IV GBM in the right frontal lobe [19]. SEBTA-027 was derived from a 59-year-old female with recurrent grade IV GBM in the right parieto-occipital region, with sarcomatous elements, wild type IDH1, MGMT is unmethylated and the original tissue was seen to have a 40% Ki67 labeling index [20]. The patient had previously received radiotherapy and Temozolomide chemotherapy. These cells were cultured in DMEM + GlutaMAX-I (Gibco, cat#: 10,566,016) supplemented with 10% v/v FBS. All cell lines were maintained in a humidified incubator at 37 °C in 5% v/v CO_2_.

### Animals and immunization schedule

C57BL/6 HHDII/DR1 transgenic mice [21] were utilized for this study. These mice express the human α1 and α2 chains of HLA-A*0201 with the α3 chain of H-2Dd allele (HHDII), also expressing HLA-DRB*0101, and knocked out for the expression of murine MHC class I (H-2b) and II (I-Ab). These were provided by Dr. Lone (CNRS, Orleans, France). Animal use and care was in accordance with EU Directive 2010/63/EU and UK Home Office Code of Practice for the housing and care of animals bred, supplied, or used for scientific purposes. The studies were undertaken with UK Home Office approval. Mice aged between 6 and 8 weeks were used for this study. SCIB1 ImmunoBody® DNA was coated onto 1.0 μm Gold Microcarriers (Bio-Rad, cat#: 1,652,263) which were subsequently administered intradermally using a Helios® gene gun (Bio-Rad). Mice were given approximately 1 µg of DNA per vaccination in the form of a priming dose on day 0 followed by a boost on day 7 and day 14, animals were terminated, and spleens removed for analysis of immune reactivity on day 21.

### Ex vivo ELISpot assay

Splenocytes were obtained by flushing 10 ml of T cell medium through the spleen, the release of IFNγ from which was then determined using a murine IFNγ ELISpot kit (Mabtech, cat#: 3321-2A). For this, MultiScreen^HTS^ IP Filter 96-well plates (Millipore, cat#: MSIPS4W10) were coated with IFNγ antibody by overnight incubation at 4 °C, after which plates were blocked by incubation with T cell medium for 30 min at room temperature. After blocking, 5 × 10^5^ splenocytes were added to triplicate wells and incubated with peptides (1 µg/mL for class I and 10 µg/mL for class II) for approximately 40 h at 37 °C in a 5% v/v CO_2_ humidified incubator. Negative control wells consisted of splenocytes alone, and positive control wells consisted of splenocytes stimulated with 2.5 µg/mL of the T cell mitogen concanavalin A (Sigma, cat#: C5275). After incubation the splenocytes were removed by washing and the captured IFNγ detected using a biotinylated IFNγ antibody and an Alkaline Phosphatase Conjugate Substrate Kit (Bio-Rad, cat#: 170–6432). Spots were counted using an ImmunoSpot Analyzer (Cellular Technology Limited).

### T cell isolation

Splenocytes from vaccinated mice were counted and T cells isolated using the Dynabeads™ Untouched™ Mouse T Cells Kit (Invitrogen, cat#: 11413D) following the manufacturer’s instructions. In brief, cells were suspended at a concentration of 1 × 10^8^ cells/mL in isolation buffer (D-PBS (Corning, cat#: 21-031-CV) + 0.1% w/v BSA (Millipore, cat#: 12,659) + 2 mM EDTA (Sigma, cat#: E6758)). 5 × 10^7^ cells were transferred to a fresh tube and 100 µL of heat inactivated FBS was added, 100 µL of antibody cocktail was added to the mixture and tubes were incubated for 20 min at 4 °C. Cells were then washed with 10 mL of isolation buffer by centrifugation at 350 × g for 8 min at 4 °C. The supernatant was discarded, and the resulting cell pellet was resuspended in 4 mL of isolation buffer. Mouse Depletion Dynabeads™ (1 mL) were added to the cell suspension and this mix was incubated at room temperature for 15 min with gentle tilting and rotation. After incubation, 5 mL of isolation buffer was added to the mixture and the beads resuspended. This tube was then placed in a magnet for 2 min and the supernatant containing T cells was poured into a fresh tube. Cells were spun down, resuspended in T cell medium and counted.

### Flow cytometry staining of TRP-2 specific T cells using a TRP-2 pentamer

Isolated T cells (1 × 10^6^) were transferred into a 12 × 75 mm polystyrene tube and washed with 2 mL of D-PBS by centrifugation at 300 × g for 5 min at room temperature. The supernatant was poured off and the resulting cell pellet was resuspended in 50 µL of FBS and 0.5 µg/µL of anti-CD16/CD32 (BioLegend, cat#: 101,302, clone 93). Tubes were incubated for 15 min at 4 °C. Cells were then incubated with 10 µL of Pro5 MHC pentamer (A*02:01/Kb SVYDFFVWL) APC conjugated pentamer (ProImmune) for 10 min at room temperature in the dark. After incubation cells were washed with 2 mL of D-PBS via centrifugation at 300 × g for 5 min at room temperature. The supernatant was removed, and the resulting cell pellet was resuspended in 50 µL of D-PBS containing 0.3 µg FITC-conjugated mouse CD45 monoclonal antibody (mAb) (Miltenyi Biotec, cat#: 130–110-658, clone REA737), 0.2 µg Brilliant Violet 421™-conjugated mouse CD3 mAb (BioLegend, cat#:100,228, clone 17A2), 0.5 µg APC/Cyanine 7conjugated mouse CD8a mAb (BioLegend, cat#:100,714, clone 53–6.7) and 0.5 µL LIVE/DEAD™ fixable yellow dead cell stain (Molecular Probes, cat#: L34959). Tubes were then incubated for 30 min at 4 °C in the dark. Cells were washed with 2 mL D-PBS via centrifugation at 300 × g for 5 min at room temperature. The supernatant was removed, and cells resuspended in 300 mL of IsoFlow Sheath Fluid (Beckman Coulter, cat#: 8,546,859), after which they were analyzed using a Backman Coulter Gallios® flow cytometer and Kaluza® acquisition and analysis software (Beckman Coulter).

### Immunocytochemical staining of B16^HHDII/DR1^ cells

Cells were grown on glass coverslips and stained once sufficiently confluent. Cells were first fixed in 4% w/v formaldehyde (Sigma, cat#: 252,549) in D-PBS for 1 h at room temperature. Cells were then washed three times with D-PBS, 5 min each time. Cells were then permeabilized with 0.1% v/v Triton X-100 (Sigma, cat#: X100) in D-PBS for 5 min at room temperature. Cells were then washed with D-PBS as before. Cells were then blocked using 2.5% v/v Normal Horse Serum (Vector Laboratories, cat#: PK-7200) for 10 min at room temperature. The serum was then removed, and cells were incubated with Avidin D solution (Vector Laboratories, cat#: SP-2001) for 15 min at room temperature. Cells were then washed in D-PBS for 2 min. Biotin (Vector Laboratories, cat#: SP-2001) was then added to cells for 15 min at room temperature. Cells were then incubated with a TRP-2 rabbit polyclonal antibody (1:150, Abcam, cat#: ab74073) or a rabbit recombinant gp100 mAb (1:250, Abcam, cat#: ab137078, clone EP4863(2)) diluted in 2.5% v/v Normal Horse Serum overnight at 4 °C. Cells were washed three times in D-PBS, cells were then incubated in Biotinylated Universal Antibody (Vector Laboratories, cat#: PK-7200) for 10 min at room temperature. Cells were then washed three times in D-PBS, 5 min each time, after which they were incubated in VECTASTAIN Elite ABC Reagent (Vector Laboratories, cat#: PK-7200) for 5 min at room temperature. Cells were washed in D-PBS as before and then incubated in DAB Substrate (Vector Laboratories, cat#: SK-4100) for 3 min. Cells were washed in distilled H_2_O for 2 min. Cells were then counterstained with Mayer’s Hematoxylin (Sigma, cat#: 51,275) and washed under tap water for 5 min. The cells on the coverslips were then mounted to slides using DPX Mountant (Sigma, cat#: 44,581) and imaged microscopically (Zeiss).

### Target cell recognition and killing

At the end of the vaccination schedule, mice were euthanized and isolated splenocytes stimulated in vitro with 0.1 µg/mL of TRP-2 peptide (SVYDFFVWL) and 50 U/mL of murine IL-2 (PeproTech, cat#: 212–12) for 5 days. CD3^+^ T cells were then isolated using an EasySep Mouse T Cell Isolation Kit (STEMCELL Technologies, cat#: 19,851), plated at 2.5 × 10^5^ cells per well and co-cultured with 1 × 10^4^ B16^HHDII/DR1^ cells per well (50:1 effector:target ratio) in the presence or absence of anti-mouse CD8a antibody at 20 mg/mL (Bio X Cell, cat#: BE0061, clone 2.43) in either an IFNγ ELISpot plate (for target cell recognition via the release of IFNγ) or a flat bottom 96-well plate (to assess target cell death). For target cell recognition, the murine IFNγ ELISpot assay was performed as described previously. For the assessment of target cell death, a WST-8 cytotoxicity assay was performed using a Cell Counting Kit-8 (CCK-8, Sigma, cat#: 96,992). WST-8 is bioreduced by cellular dehydrogenases to an orange formazan product that is soluble in tissue culture medium. The amount of formazan produced is directly proportional to the number of living cells. After plating the cells in a final volume of 200 µL per well, the plate was incubated for 24 h at 37 °C in a 5% v/v CO_2_ humidified incubator. Control wells included medium alone as a blank; T cells alone and target cells alone to account for spontaneous cell death; and target cells with 0.05% w/v SDS as a death control. 20 µL of CCK-8 solution were then added to each well and incubated for 4 h in the incubator and the absorbance was read at 450 nm using an iMark Microplate Absorbance Reader (Bio-Rad).

### *PD-L1* and *IDO1* expression in low grade glioma (LGG) and GBM

The expression of *PD-L1* and *IDO1* in LGG and GBM, as well as the expression of *CD4, CD39, CD73, TIM3, IL-10* and *TGFB1* in GBM were assessed using Gene Expression Profiling Interactive Analysis 2 (GEPIA 2, http://gepia2.cancer-pku.cn/#index) [22], a web server for gene expression analysis from healthy and tumor samples obtained from the TCGA and the GTEx projects. GEPIA 2 holds data for 207 normal brain samples from the GTEx project as well as 518 LGG samples and 163 GBM samples from the TCGA project.

### Intracranial tumor implantation

All animal work was carried out under a Home Office approved project license and reviewed by the Nottingham Trent University’s Animal Welfare Ethical Review Body (AWERB). The animals were housed in groups of three in Techniplast Sealsafe Plus cages and furnished with corncob bedding, chew sticks, sizzle-nest and play tunnels (Datesand Ltd., Cheshire, UK). The animals were allowed free access to food and water. A pelleted diet (Rodent 2018C Teklad Global Certified Irradiated Diet, Envigo Laboratories U.K. Ltd., Oxon, UK) was used. Mains drinking water was supplied from polycarbonate bottles attached to the cage. The diet and drinking water were considered not to contain any contaminant at a level that might have affected the purpose or integrity of the study. They were housed in a single air-conditioned room within the Bioscience Support Facilities at Nottingham Trent University, Nottingham, UK. The rate of air exchange was at least fifteen air changes per hour and the low intensity fluorescent lighting was controlled to give twelve hours continuous light and 12 h darkness. Environmental conditions were continuously monitored by a computerized system. The temperature and relative humidity controls were set to achieve target values of 22 ± 2 °C and 55 ± 15%, respectively. Deviations from these targets were considered not to have affected the purpose or integrity of the study. The animals were allocated to treatment groups as they came to hand. These were uniquely identified within the study by an ear punching system routinely used in these laboratories.

Prior to surgery all animals had lidocaine applied on the scalp overlying the proposed craniotomy site. Buprenorphine was administered at a dose of 0.05–0.1 mg/kg s/c immediately prior to surgery and thereafter every 12 h if required. Carprofen was administered at a dose of 5 mg/kg subcutaneously immediately after surgery before recovery and thereafter every 24 h if required. Postoperatively, all animals were kept warm at 37 °C in warming box and monitored until they regained consciousness and were able to stand.

All animals were anaesthetized using isoflurane (Zoetis) at a flow rate of approximately 4 L/min. The anesthetic was maintained at 2% for the duration of the surgery. Stereotaxic intracranial implantation of B16^HHDII/DR1^ cells was performed by injecting 4–5 × 10^3^ cells in 3 μL D-PBS into the striatum, avoiding the sagittal sinus vessel (2 mm lateral, right-hand side, 1 mm anterior to bregma and at a depth of 3.5 mm from the cortical surface). After 60 s the needle was withdrawn to depth of 3 mm and then cells were injected at a rate of 1.5 μL per 60 s, over 2 min. After implantation the needle was left in place for 60 s and then withdrawn at a rate of 0.1 mm every 2 s until clear of the brain. Once the injection was completed the wound was closed and mice were monitored at regular intervals.

#### Immunization schedule for tumor bearing mice

Mice harboring intracranial tumors were vaccinated with SCIB1 ImmunoBody® (*n* = 10) or sham vaccinated (*n* = 5) using an empty gene gun bullet on days 3, 7, 10, 17, 24 and 31 post tumor implantations. Mice were then given either 250 µg of PD-1 antibody (*n* = 10) (clone RMP1-14, Bio X Cell) or 250 µg of isotype control antibody (for the sham vaccinated group) (anti-Trinitrophenol clone 2A3, Bio X Cell) in a volume of 100 µL (in sterile D-PBS) intraperitoneally on days 4, 8, 11, 18, 25 and 32 post tumor implants. Mice were monitored at regular intervals twice a day, once a day at the weekend. All animals were body weighed daily and humanely culled if they displayed any of the following: Body weight loss of ≥ 18% in the first 5 days post-surgery and after 5 days post-surgery, a body weight loss of up to 15% in combination with other clinical signs, indicative of a deterioration in the physical condition of the animal or body weight loss exceeding 15% and observed in isolation. Animals were also culled if disturbances to the normal behavior repertoire such as hunched posture, subdued behavior, piloerection were observed.

#### Immunofluorescence

Mouse brains were harvested and fixed in 4% w/v formaldehyde at 4 °C overnight, placed in 30% w/v sucrose at 4 °C until they sunk to the bottom of the tube and then frozen-embedded in OCT compound (Scigen, cat#: 51-1625-0019); 3 brains per treatment group (*n* = 3) were then cryosectioned to 30 µm slices and mounted on Epredia SuperFrost Plus slides (Fisher Scientific, cat#: 12312148PBS); 3–4 brain slices were mounted per slide to allow for technical replicates. The slices were washed three times with D-PBS, 5 min each time. They were then washed with 0.2% v/v Triton X-100 in D-PBS for 5 min followed by a 1-h blocking step with blocking buffer composed of 5% w/v BSA diluted in 0.2% v/v Triton X-100 in D-PBS. Staining was done over a 3-day period at 4 °C using the following primary antibodies diluted in blocking buffer: polyclonal guinea pig anti-mouse CD4 polyclonal antibody (1:500, Synaptic Systems, cat#: HS-360 004) and rat anti-mouse CD8a mAb (1:400, Abcam, cat#: ab22378, clone YTS169.4). Sections were washed three times with D-PBS, 5 min each time, and then incubated for 2 h with the following secondary antibodies diluted in blocking buffer: Alexa Fluor™ 488-conjugated goat anti-guinea pig IgG (H + L) (1:1,000, Invitrogen, cat#: A-11073) polyclonal antibody and Alexa Fluor™ 633-conjugated goat anti-rat IgG (H + L) (1:1,000, Invitrogen, cat#: A-21094). Sections were washed twice with D-PBS, 5 min each time, and then washed once with 20 µg/mL of DAPI (Sigma) diluted in distilled H_2_O for 5 min. Sections were then mounted using VECTASHIELD Antifade Mounting Medium (Vector Laboratories, cat#: H-1000–10), imaged using a Leica SP5 confocal microscope and images analyzed using ImageJ [23] following the ’Automated cell counting from tissue sections’ quantification method described by Shihan and colleagues [24], replacing the auto threshold option ‘Otsu’ with ‘Yen’ for optimal thresholding.

## Statistical analysis

All statistical analyses were performed using GraphPad Prism 8 software. The statistical test applied and the significance are indicated within the figure legends.

## Results

### SCIB1 ImmunoBody® generates a strong anti-TRP-2 immune response in HHDII/DR1 mice

In silico analysis using the SYFPEITHI database [25] revealed the predicted MHC binding scores of the peptides encoded by the SCIB1 vaccine (Table 1). The higher the score, the stronger the predicted binding of the peptide to the HLA allele indicated. This method was used to select the peptides used for the in vitro stimulation of splenocytes from vaccinated HHDII/DR1 humanized mice.

**Table 1:** Peptides used to stimulate vaccinated splenocytes and their predicted MHC binding scores using SYFPEITHI [25]

Peptide	Amino acid sequence	Human HLA-specificity	SYFPEITHI score
TRP-2 SVY	SVYDFFVWL	A2 (HHDII)	21
gp100 QLY	QLYPEWTEA	A2 (HHDII)	19
gp100 AML	AMLGTHTMEV	A2 (HHDII)	26
gp100 NRQ	NRQLYPEWTEAQRLD	DR1	14
gp100 GTG	GTGRAMLGTHTMEVT	DR1	24

**Fig. 1:**
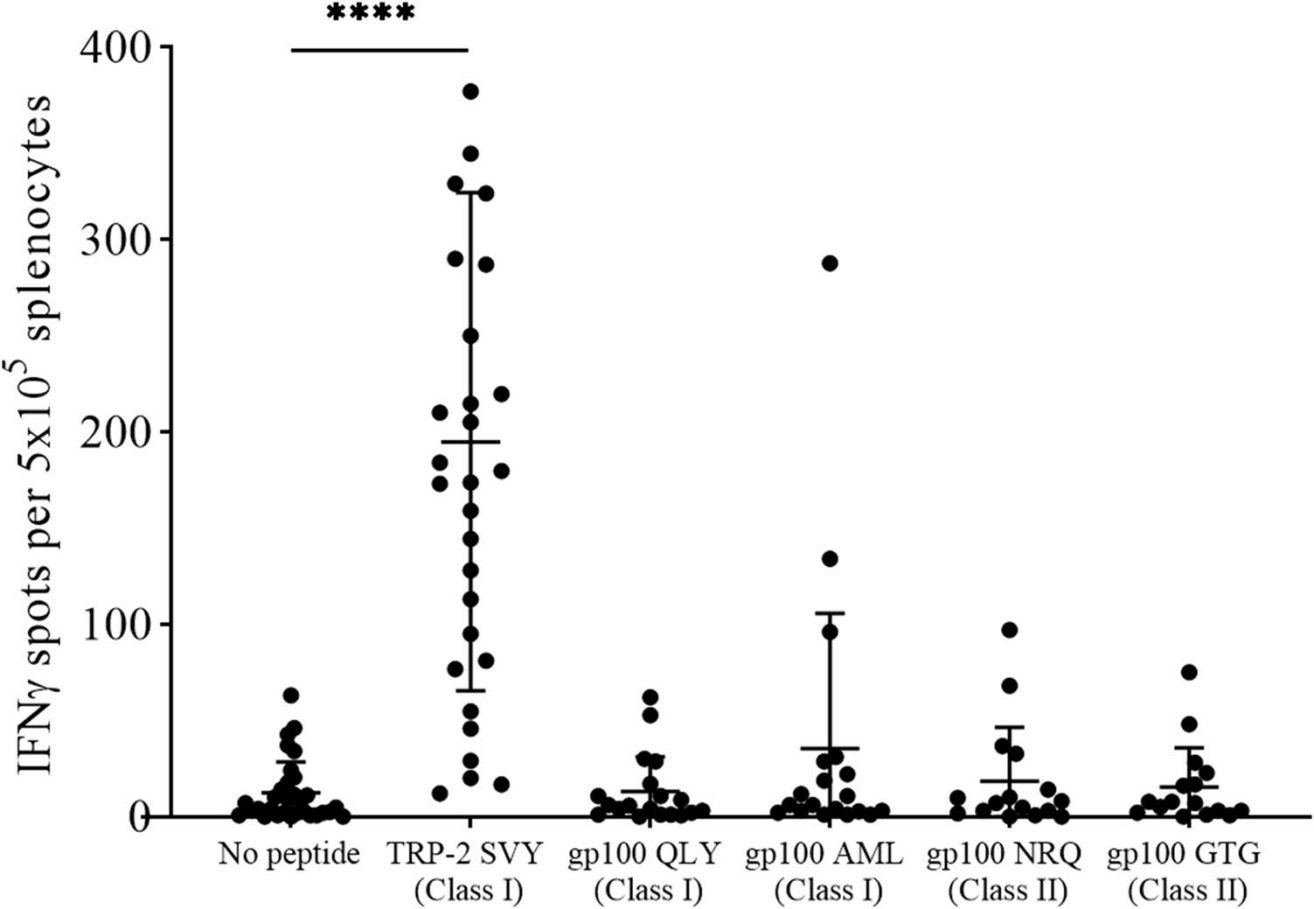
ELISpot results from *in vitro* stimulated splenocytes from SCIB1 ImmunoBody.® vaccinated C57BL/6 HHDII/DR1 mice. **** *p* ≤ 0.0001 as determined by a Kruskal–Wallis test followed by Dunn’s multiple comparisons test (*n* = 16–31)

To determine the immunogenicity of the SCIB1 ImmunoBody® vaccine, C57BL/6 HHDII/DR1 mice were vaccinated with the SCIB1 ImmunoBody® on day 0, 7 and 14 in a prime, boost, boost schedule and on day 21 the spleens were harvested and the splenocytes were then stimulated with the peptides identified in Table 1. The response to these peptides was measured using an IFNγ ELISpot assay. SCIB1 ImmunoBody® vaccination generated a strong TRP-2 specific response, indicating that the TRP-2 peptide sequence within the ImmunoBody® construct was endogenously processed and presented to T cells resulting in the observed IFNγ release. Although several mice also generated responses to the gp100 peptides, these were at a much lower frequency than the TRP-2 responses observed (Fig. 1). The reduced frequency of responses to the gp100 peptides may be due to epitope dominance of the TRP-2 sequence, which is a well-known dominant sequence [26]. Moreover, due to the HLA-restriction of the mice used here, it is entirely possible that the full T cell helper repertoire which could be generated via other MHC class-II gp100 derived peptides was not generated compared to what can be achieved with the same vaccine administered in humans [11].

**Fig. 2:**
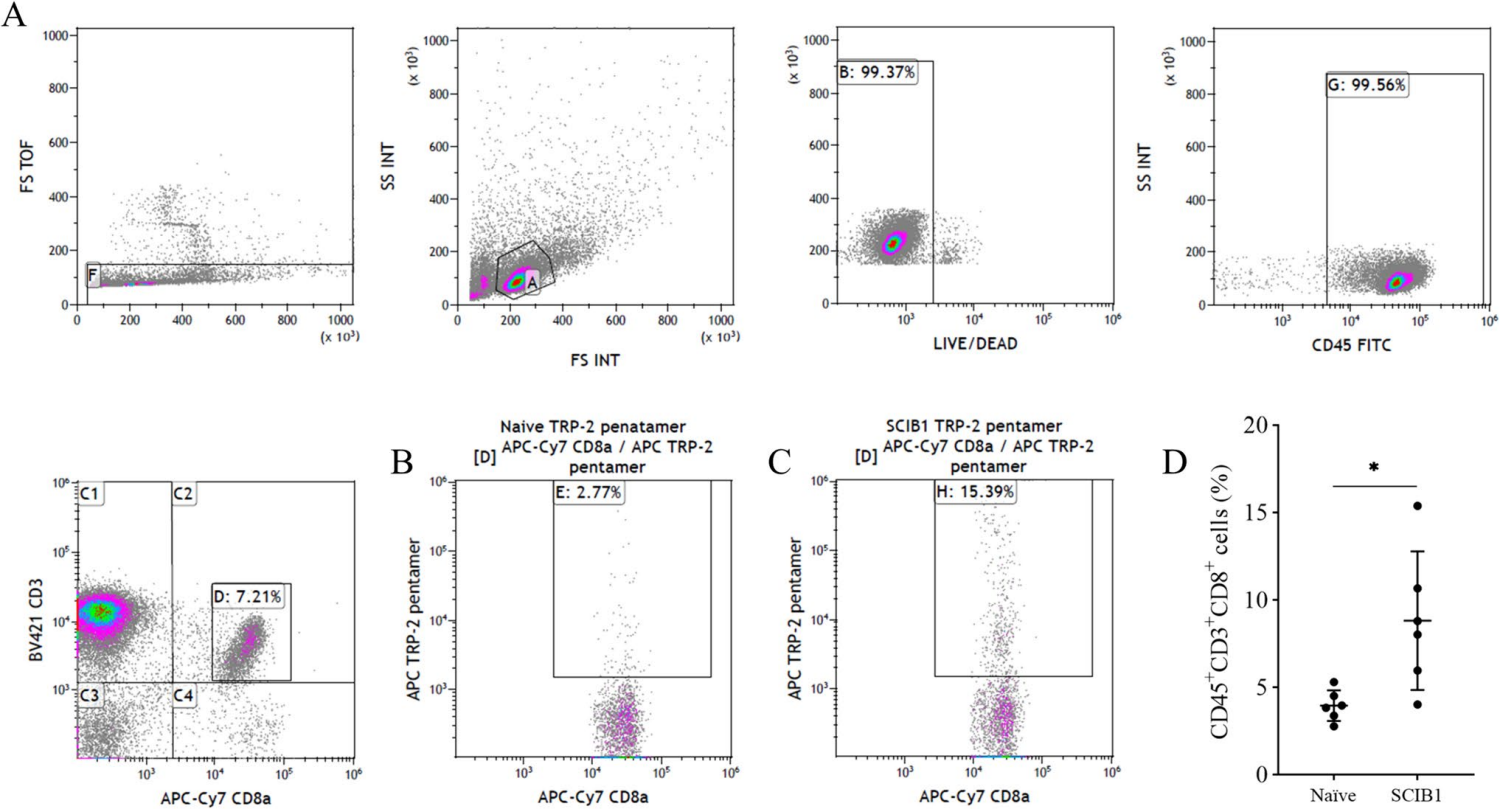
TRP-2 pentamer positive cells in CD3^+^ cells isolated from SCIB1 ImmunoBody® vaccinated mice. **A** Example of the gating strategy used to analyze the TRP-2 pentamer staining with example plots from, **B** naïve splenocytes and **C** SCIB1 ImmunoBody® vaccinated splenocytes. **D** Percentage of pentamer positive CD45^+^, CD3^+^, CD8^+^ splenocytes from naïve and SCIB1 ImmunoBody.® vaccinated mice. * *p* ≤ 0.05 as determined by an unpaired *t* test (*n* = 6)

To further examine the presence of TRP-2 specific CD8^+^ T cells, TRP-2 pentamer staining was performed on CD3^+^ cells isolated from SCIB1 ImmunoBody® vaccinated and naïve mice. Pentamer staining revealed a significant increase in the percentage of TRP-2 specific CD8^+^ T cells in the spleens of SCIB1 ImmunoBody® vaccinated mice when compared to unvaccinated naïve mice (Fig. 2). These data demonstrate that vaccination increases the percentage of CD8^+^ T cells specific for the TRP-2 180–188 amino acid sequence, further supporting the IFNγ ELISpot results reported.

**Fig. 3:**
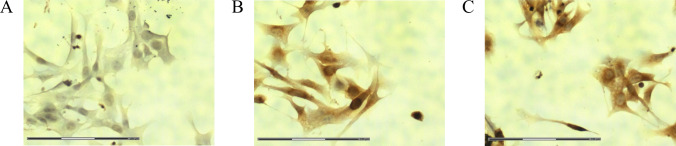
Immunocytochemical staining of the B16^HHDII/DR1^ cell line. **A** TRP-2 staining, **B** gp100 staining, **C** No primary antibody control. Positive staining is indicated by brown coloration. Scale bar = 150 µm

We have shown that SCIB1 ImmunoBody® generates a strong immune response which resulted in the recognition of the TRP-2_180–188_ sequence, a sequence which has previously been shown to be vital for the rejection of murine melanoma [26]. B16 cells are well-known to express both TRP-2 [26, 27] and gp100 [28, 29], and our own analyses revealed that B16^HHDII/DR1^ cells also express TRP-2 and gp100 (Fig. 3).

**Fig. 4:**
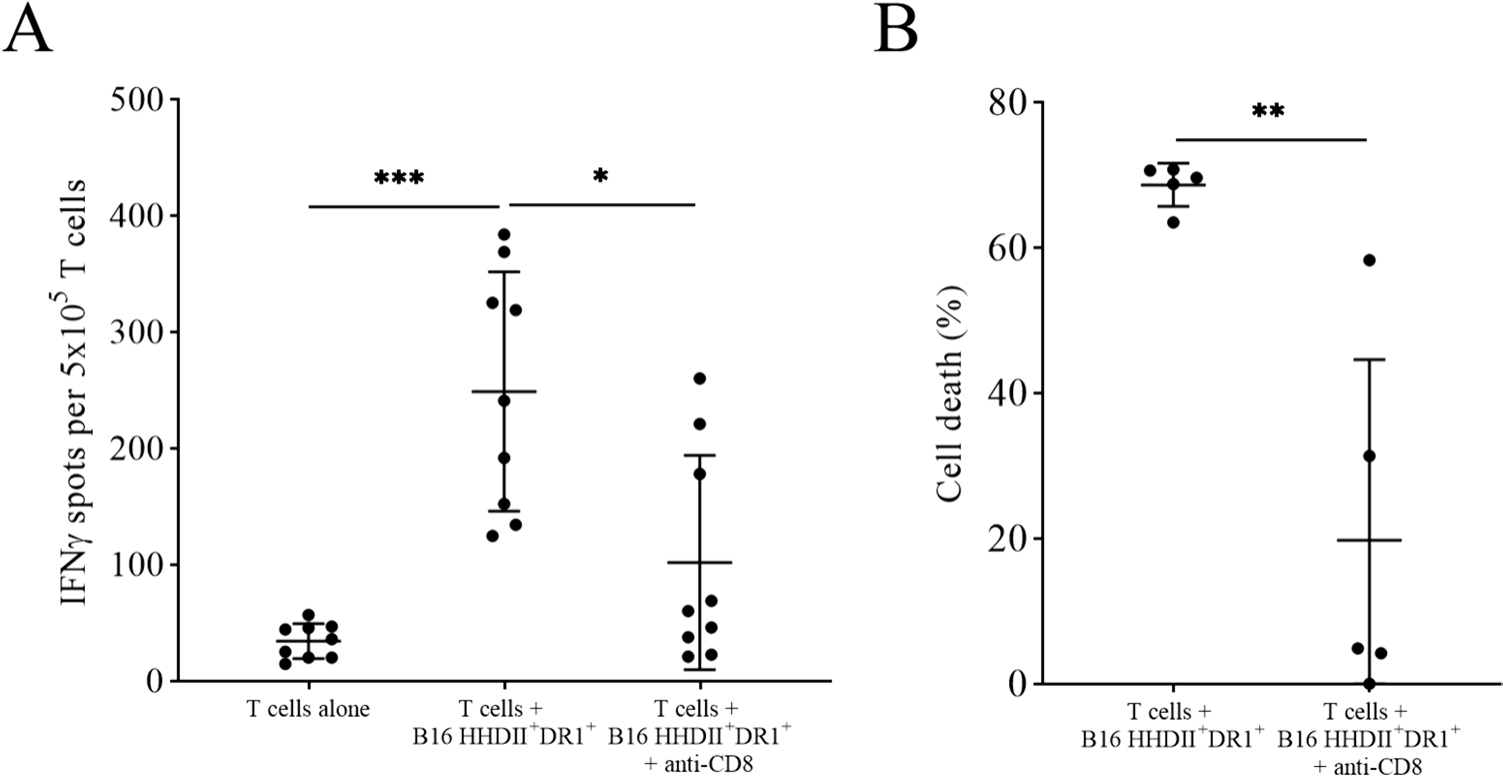
Vaccine-induced T cell-specific B16^HHDII/DR1^ recognition and killing after short-term *in vitro* culture with TRP-2 peptide and IL-2. Splenocytes of immunized animals were cultured for 7 days *in vitro* in the presence of low levels of TRP-2 and IL-2. Seven days later, CD3^+^ T cells were isolated and co-cultured with B16.^HHDII/DR1^ cells at an E:T ratio of 50:1 in the presence or absence of anti-mouse CD8a antibody in either an IFNγ ELISpot plate to assess target recognition (**A**) or in a flat bottom 96-well plate to assess target killing using a WST-8 cytotoxicity assay (**B**). For the IFNγ ELISpot, * *p* ≤ 0.05 and *** *p* ≤ 0.001 as determined by a Friedman test followed by Dunn’s multiple comparisons test (*n* = 9). For the WST-8 cytotoxicity assay, ** *p* ≤ 0.01 as determined by a paired *t* test (*n* = 5)

B16^HHDII/DR1^ cells were then used as target cells in a co-culture assay with T cells isolated from the splenocytes from immunized mice that had been cultured in vitro with TRP-2_180–188_ peptide and IL-2. The results showed that the T cells from vaccinated mice recognize B16^HHDII/DR1^ cells in a CD8-specific manner (Fig. 4A) by releasing IFNγ and trigger their cytotoxicity, as assessed by a WST-8 cytotoxicity assay (Fig. 4B).

Although checkpoint inhibitors have revolutionized the treatment for some cancers such as melanoma, not all types of cancer respond due to their lack of PD-L1 expression, Furthermore, some cancers face additional hurdles due to the immunosuppressive effect of indoleamine-2,3-dioxygenase (IDO) expression. We therefore assessed whether GBM would lend itself for such a therapy in combination with active immunotherapy.

**Fig. 5:**
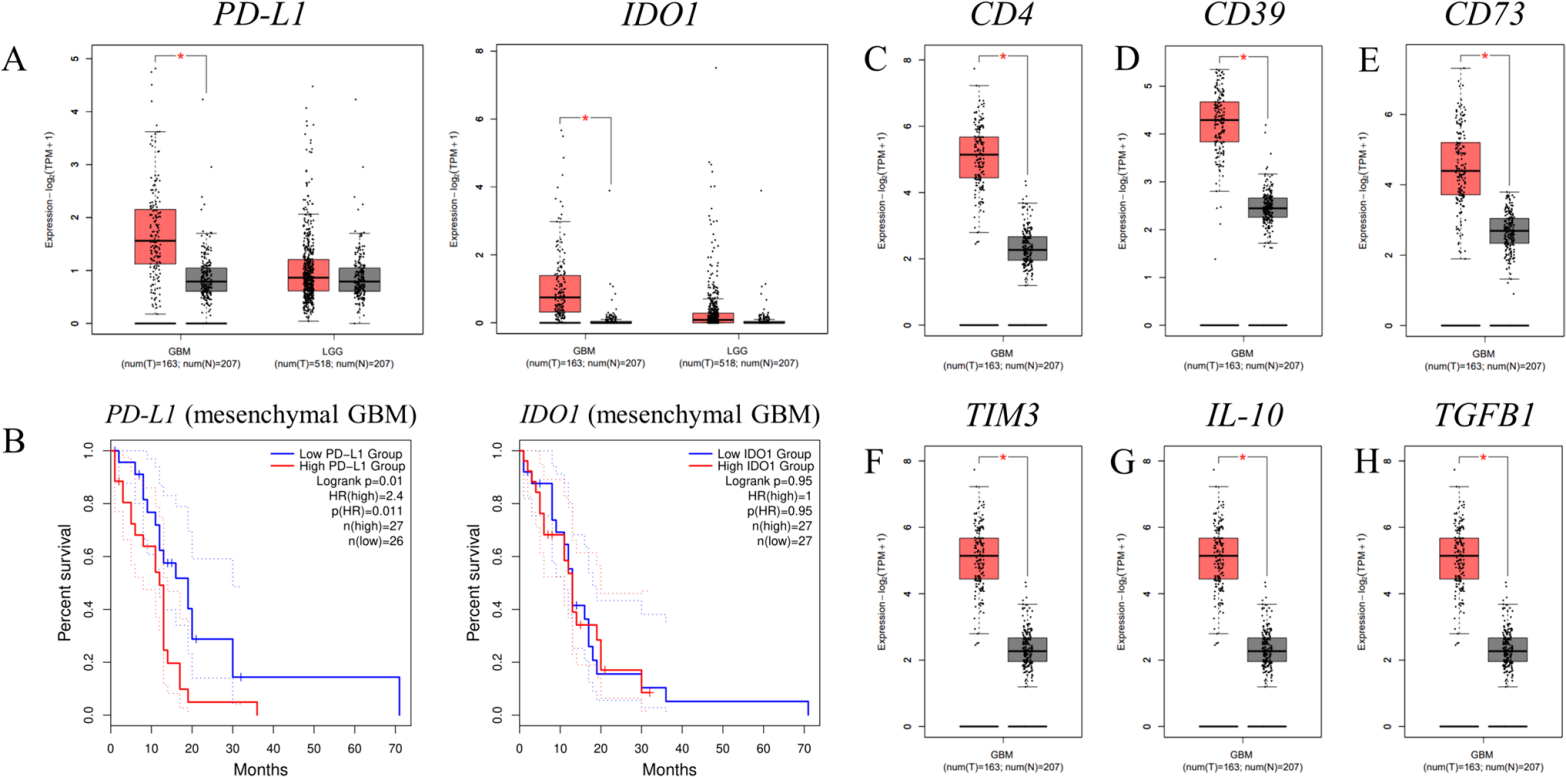
GEPIA2 gene analysis of immunosuppressive gene expression in low grade glioma (LGG) and GBM. **A** *PD-L1 and IDO1* expression is shown in healthy samples compared with LGG and GBM samples; * *p* ≤ 0.01 as determined by one-way ANOVA and log2FC cutoff value of 0.7. **B** Kaplan Meier survival curves are also shown for both genes; *p* = 0.011 and *p* = 0.95 as determined by a Log-rank (Mantel–Cox) test. **C** *CD4*, **D** *CD39*, **E** *CD73*, **F** *TIM3*, **G** *IL-10* and **H** *TGFB1* expression is shown in healthy samples compared to GBM samples; * *p* ≤ 0.01 as determined by one-way ANOVA and log2FC cutoff value of 1

The Gene Expression Profiling Interactive Analysis database (GEPIA2, http://gepia2.cancer-pku.cn) revealed a significantly higher expression of *PD-L1* and *IDO1* transcripts in GBM patient samples compared to normal tissue, and in both cases the difference in expression was marked when compared to low grade glioma (LGG) (Fig. 5A). Patients with high expression of *PD-L1* also have a significantly lower overall survival than those with a low expression of the molecule (albeit only for the mesenchymal subtype of GBM) (Fig. 5B).

On the basis of these findings, we combined SCIB1 ImmunoBody® with PD-1 checkpoint inhibition.

### SCIB1 ImmunoBody® vaccination in combination with PD-1 blockade prolongs the time-to-death of mice bearing intracranial B16^HHDII/DR1^ tumors and promotes T cell tumor infiltration

**Fig. 6:**
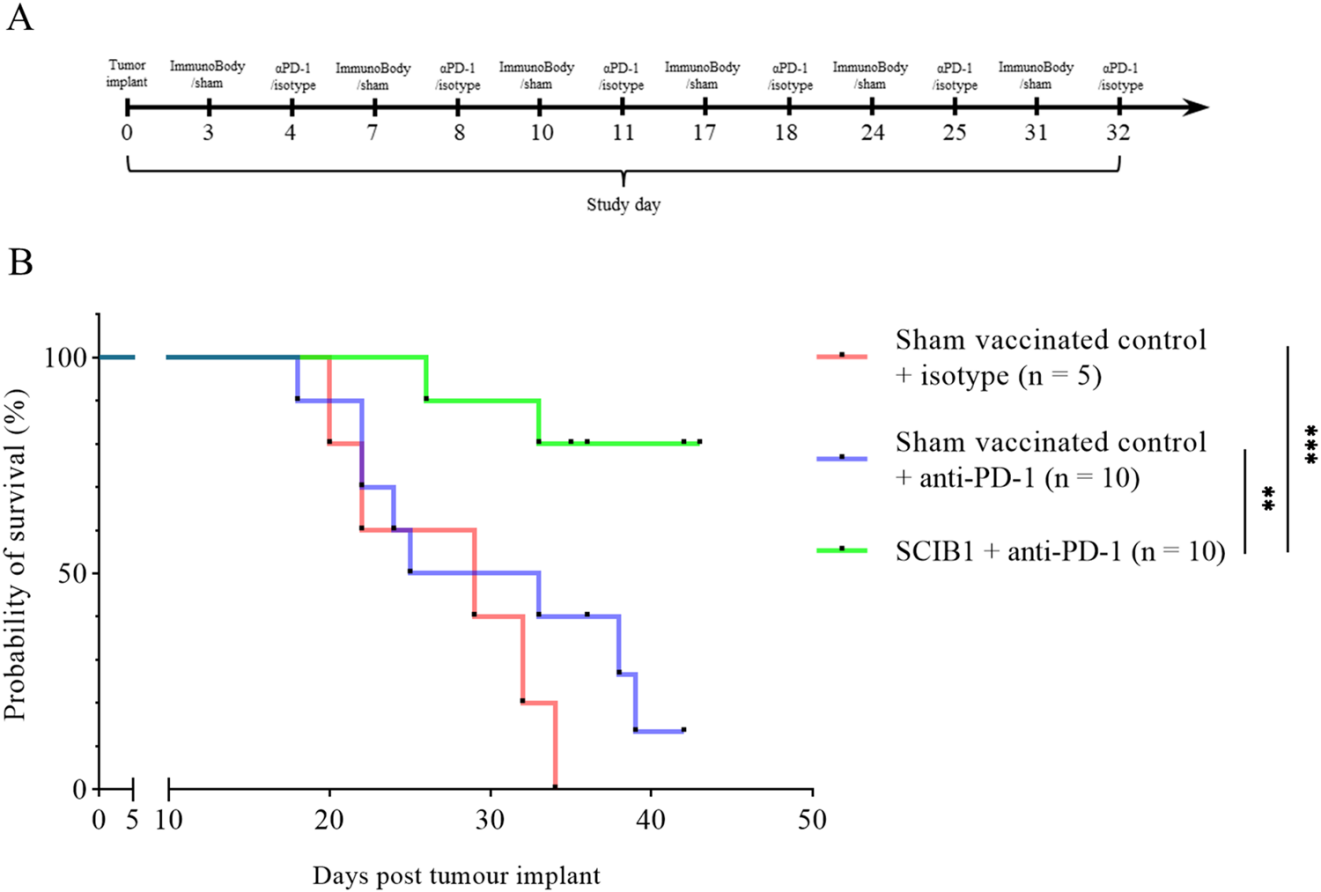
**A** Schematic representation of the therapeutic dosing regimen utilized to treat intracranial B16^HHDII/DR1^ tumors. **B** Kaplan Meier survival curve of mice bearing intracranial B16.^HHDII/DR1^ Luc tumors. ** *p* ≤ 0.01; *** *p* ≤ 0.001 as determined by a Log-rank (Mantel–Cox) test (*n* = 5 for sham vaccinated mice; *n* = 10 for sham + PD-1 as well as SCIB1 + PD-1 treatment groups)

**Fig. 7:**
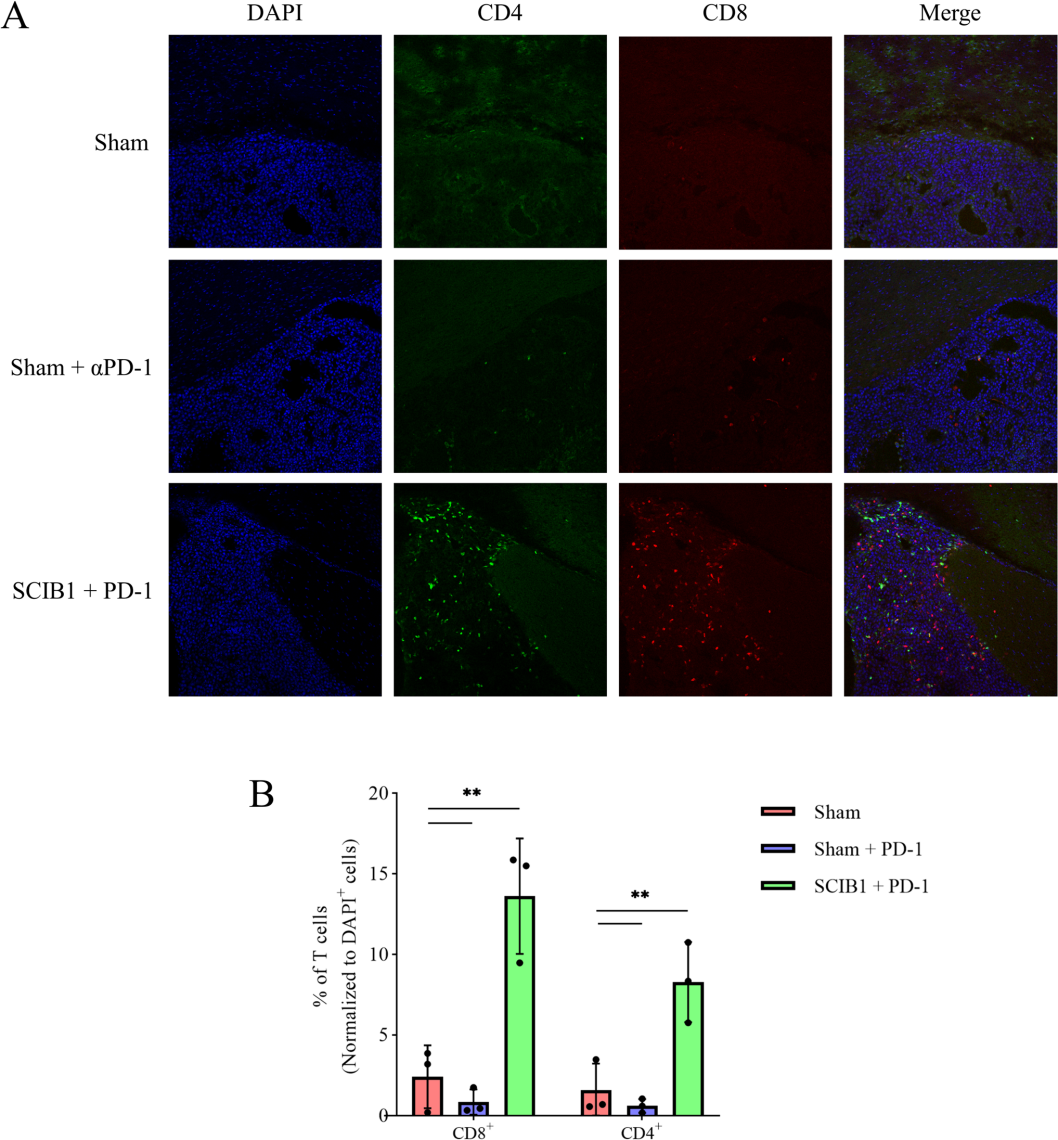
Immunofluorescence staining and quantification of CD4^+^ and CD8^+^ T cell tumor infiltration following SCIB1 ImmunoBody® vaccination. The cryosectioned brains of sham, sham + anti-PD-1 and SCIB1 ImmunoBody® + anti-PD-1 vaccinated mice were stained for CD4 and CD8a and imaged to study T cell tumor infiltration (**A**); the acquired images were processed using ImageJ to quantify CD4^+^ and CD8.^+^ T cells within the tumor area (**B**). ** *p* ≤ 0.01 as determined by a one-way ANOVA followed by Tukey’s multiple comparisons test (*n* = 3)

B16^HHDII/DR1^ cells were injected into the brains of C57BL/6 HHDII/DR1 mice. These mice were then vaccinated with SCIB1 ImmunoBody® or sham vaccinated on days 3, 7,10, 17, 24 and 31 post tumor implantation. Mice were also given 250 µg PD-1 mAb or isotype control antibody on days 4, 8, 11, 18, 25 and 32 post tumor implantation. Mice bearing intracranial B16^HHDII/DR1^ cells showed prolonged survival when receiving combined SCIB1 and anti-PD-1 therapy after tumor implantation (Fig. 6). PD-1 mAb treatment had a modest time-to-death benefit when administrated alone compared to sham vaccinated mice that received an isotype control antibody. These data suggest that the effect of PD-1 blockade on survival was modest; whereas, the addition of SCIB1 ImmunoBody® vaccination to PD-1 blockade resulted in a significantly improved time-to-death gap of mice harboring intracranial tumors. This correlated with a significantly higher number of CD4^+^ and CD8^+^ T cells within the tumor micro-environment in the combined therapy compared to both sham and sham + anti-PD-1 treatment groups (Fig. 7).

These proof-of-concept experiments revealed that combining SCIB1 ImmunoBody® and PD-1 blockade represents an attractive therapeutic option for intracranial tumors expressing TRP-2 and, to a lesser extent, gp100.

## Discussion

Intracranial tumors present a unique therapeutic challenge due to their physiological location; and while, the brain was previously thought to be an immunoprivileged site, there is increasing evidence which suggests that this is not the case and active immune responses have now been reported [30–32]. Primary brain tumors, especially GBM tumors, have previously been shown to express both the TRP-2 and gp100 melanoma antigens due to the shared neuroectodermal origin of both tissue types [12–14]. Although expression is not ubiquitous, a large proportion of tumors express at least one of these antigens, making them attractive targets for the immunotherapeutic treatment of GBM. Our analyses of GBM tissue microarrays (TMAs) revealed that 22/33 (66%) of cases studied express the TRP-2 antigen as indicated by IHC (Supplementary Fig. 1). The expression level varied among the cases studied, with 1/33 (3%) having strong staining, 4/33 (12%) having moderate staining and 17/33 (52%) having low levels of staining. GBM tissues were also stained with an antibody directed against gp100, however only weak staining was observed in 1/33 (3%) of cases and the other 32 cases were negative (data not shown). Other research has found that a high proportion of GBM cell lines and tissues express gp100 [13, 14]; however, the samples included in our tissue microarray did not appear to have the same level of gp100 expression.

The work presented here made use of C57BL/6 mice which have been engineered to express no murine MHC molecules but instead express chimeric HLA-A2 and human HLA-DR1 molecules [21]. Using these mice, we were able to show that SCIB1 ImmunoBody® vaccination generated a strong TRP-2 specific immune response in vaccinated mice. SCIB1 ImmunoBody® has already been shown to generate stronger, and more potent immune responses than other vaccine methods such as peptides or peptide-pulsed DCs [7].

Here we show that this method of vaccination resulted in a high frequency of TRP-2 specific CD8^+^ T cells. More importantly, we showed that vaccine-induced T cells isolated after a short in vitro culture could recognize and kill B16^HHDII/DR1^ cells in a CD8^+^ dependent manner. Since GBM tumors express high levels of PD-L1, which was shown to be linked to shorter survival, we decided to combine SCIB1 ImmunoBody® vaccination with PD-1 blockade to assess the time-to-death of humanized C57BL/6 HHDII/DR1 mice harboring intracranial B16^HHDII/DR1^ tumors expressing TRP-2 and gp100. While this model is far from ideal due to the use of heavily modified tumor cells inserted directly in the brain of the mice, it allows us to investigate whether a vaccine such as SCIB1, which has already demonstrated great clinical benefit in malignant melanoma patients [11] delivered systemically, could produce a clinically relevant outcome for a tumor located in the brain. We have been able to demonstrate that immune cells activated using the SCIB1 ImmunoBody® vaccination can exert therapeutic efficacy in an intracranial tumor model prolonging the time-to-death of mice when combined with anti-PD-1 immune checkpoint blockade. This intracranial B16^HDDII/DR1^ tumor model acts as a proof-of-concept for targeting intracranial tumors expressing TRP-2 and gp100.

Importantly, the ImmunoBody® construct can also be engineered so that other antigens can be targeted, with constructs encoding the Wilms tumor 1 (WT1) and HAGE antigens showing efficacy in vivo [33, 34]. A combinatorial WT1 and HAGE ImmunoBody® vaccine demonstrated efficacy against tumors expressing HAGE and WT1 antigens [34]. In addition, it has also been shown that these antigens are expressed by GBM tumors and cell lines [35–37].

The targeting of multiple antigens will help to overcome the development of immune escape variants, enabling long-term tumor eradication. However, a successful vaccine will induce the production of IFNγ, and this cytokine is able to actively upregulate expression of PD-L1 on the surface of many cells, including the GBM cell lines we tested (Supplementary Fig. 2), making the blockade of the PD-1/PD-L1 pathway an attractive adjunct for SCIB1 ImmunoBody® immunotherapy. Furthermore, other analyses have revealed that PD-L1 expression is observed in GBM tumor tissues, and this expression is linked with poorer patient survival [38, 39]. Importantly, PD-L1 is not the only immunosuppressive protein upregulated in response to IFNγ. Infiltrating T cells have been shown to increase IDO expression in GBM and contribute to a decreased patient survival [40]; we have also found that both SEBTA-027 and SF-188 cell lines upregulated immunosuppressive IDO in response to IFNγ (Supplementary Fig. 3) and we are currently investigating this in our model. This in turn was shown to increase the recruitment of immunoregulatory T (Treg) cells and decrease overall survival in experimental mice with brain tumors [41]. Furthermore, we have found that the TME of GBM patients has significantly higher levels of *CD4*, *CD39*, *CD73*, *TIM3*, *IL-10* and *TGFB1* transcripts, pointing toward high levels of Treg cells (Fig. 5C–H). It would therefore be of particular interest to test the possibility of reprogramming these Treg cells into effector T cells with the combined use of αGITR and αPD-1 antibodies [42].

In summary, we propose that active immunotherapy in the form of SCIB1 ImmunoBody® therapy should next be assessed with both αGITR and αPD-1 antibodies or with additional means to relieve T cell exhaustion, as this could lead to an enhanced survival outcome.

### Supplementary Information

Below is the link to the electronic supplementary material.

Supplementary file1 (DOCX 1126 KB)

Supplementary file2 (PDF 1450 KB)

## Data Availability

All data generated or analyzed during this study are included in this published article [and its supplementary information files].
