# Supplementary material for: TRP-2 / gp100 DNA vaccine and PD-1 checkpoint blockade combination for the treatment of intracranial tumors
Source: Cancer Immunol Immunother. 2024 Jul 2;73(9):178. doi: 10.1007/s00262-024-03770-x (PMC11219641; doi:10.1007/s00262-024-03770-x)
Supplement: Supplementary file 1 — Supplementary file1 (DOCX 1126 KB) [file 262_2024_3770_MOESM1_ESM.docx]

Supplementary Material

TRP-2 / gp100 DNA vaccine and PD-1 checkpoint blockade combination for the treatment of intracranial tumors

Joshua R. D. Pearson, Carles Puig-Saenz, Jubini E. Thomas, Lydia D. Hardowar, Murrium Ahmad, Louise C. Wainwright, Adam M. McVicar, Victoria A. Brentville, Chris J. Tinsley, A. Graham Pockley, Lindy G. Durrant, Stephanie E. B. McArdle*

*** Correspondence:** Stephanie E. B. McArdle: stephanie.mcardle@ntu.ac.uk


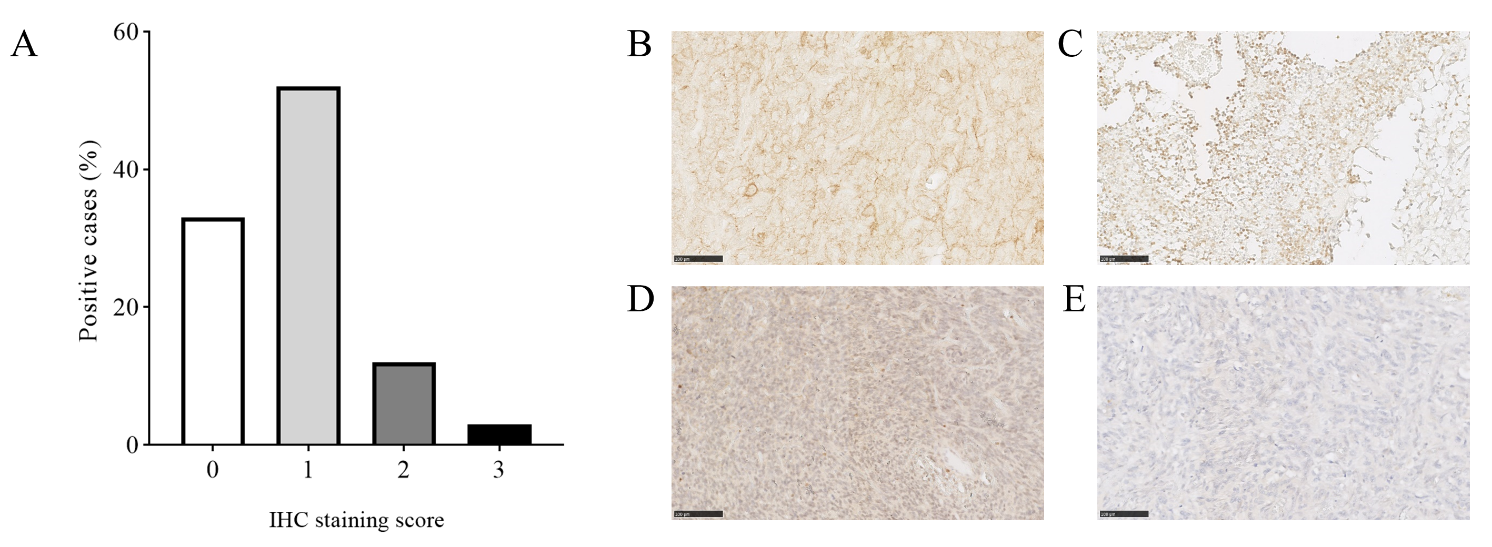


**Supplementary Figure 1.** TRP-2 protein expression in GBM tissues (A) including examples of strong (B), moderate (C), weak (D) and no staining (E). 33 cores from a GBM tissue microarray slide (US Biomax, cat#: GL806c) were stained for TRP-2 by following a similar protocol to that of ‘Immunocytochemical staining of B16^HHDII/DR1^ cells’ (main Materials and Methods section). The slide was baked at 60°C for 30 minutes, dewaxed by two consecutive immersions in xylene (4 minutes each) and then rehydrated by immersion in graded ethanol (100%, 100%, 90% and 70%; 5 minutes each). After immersion in distilled H_2_O for 2 minutes, endogenous peroxidase activity was blocked using 3% v/v hydrogen peroxide (H_2_O_2_) in methanol. The slide was rinsed in distilled H_2_O for 3 minutes and then immersed in antigen retrieval buffer (Tris/EDTA pH 9.0) at ~100°C for 20 minutes. The slide was left in the antigen retrieval buffer to cool for 20 minutes and washed in TBS-T for 5 minutes before blocking with 2.5% v/v normal horse serum. The aforementioned immunocytochemistry protocol was then followed with the following exceptions: washes were performed with 2 x 10-minute rounds of TBS-T (instead of 3 x 5-minute rounds of D-PBS); primary TRP-2 antibody was diluted 1:500 and left for 1 hour at room temperature; and before mounting the coverslips, the slide was dehydrated by consecutive immersions in 70%, 90%, 100% and 100% v/v ethanol and then finally twice in xylene. Once dry, the slide was imaged using a Hamamatsu NanoZoomer digital slide scanner. Our analysis revealed that 22/33 (66%) of cases express TRP-2. The expression level was varied amongst the cases studied with 1/33 (3%) having strong staining, 4/33 (12%) having moderate staining and 17/33 (52%) having weak staining.


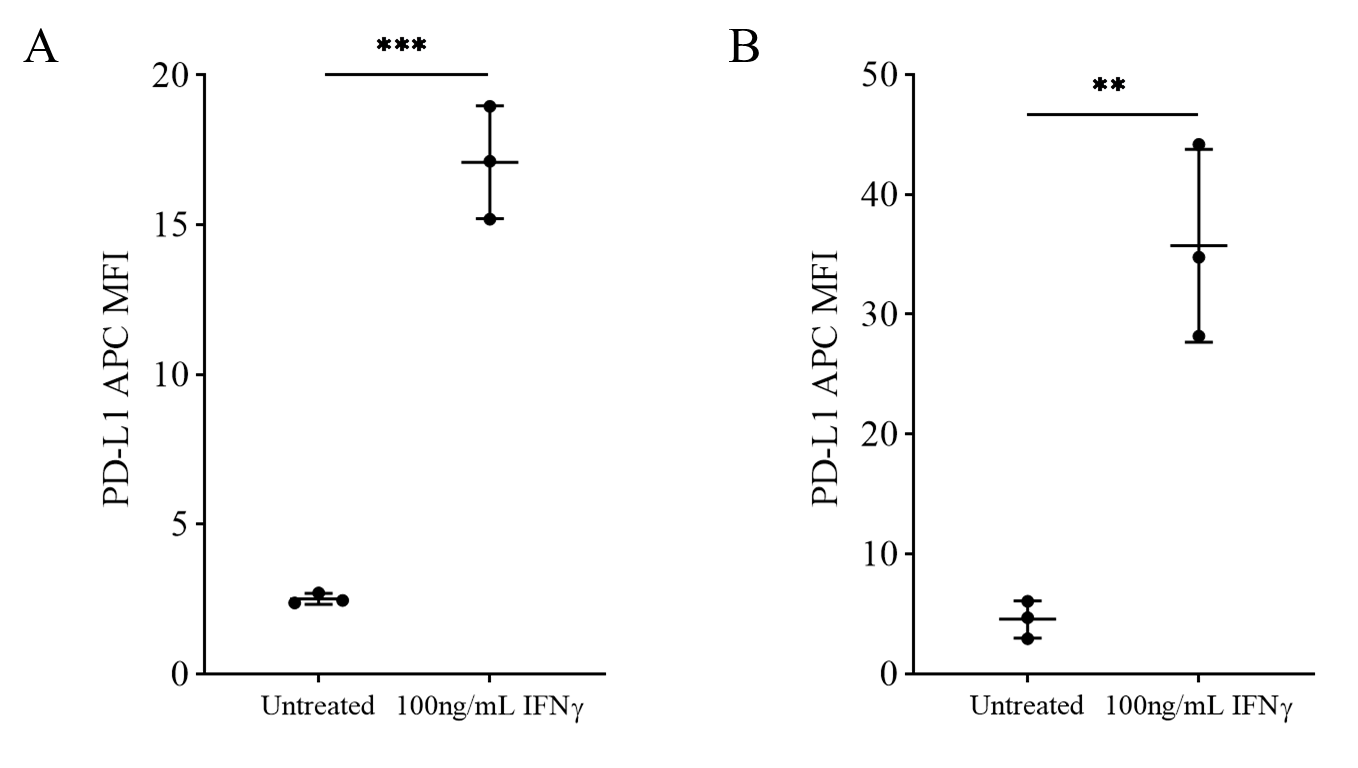


**Supplementary Figure 2.** SEBTA-027 (A) and SF-188 (B) GBM cell lines actively respond to IFNγ by upregulating PD-L1 on their surface. Cells were treated with 100 ng/mL of human IFNγ (Peprotech) for 72 hours. 1x10^6^ cells were washed with 2 mL of D-PBS by centrifugation at 300 x g for 5 minutes. The supernatant was poured off and the resulting cell pellet was re-suspended in 50 µL of FCS containing 0.5 µg/µL of anti-CD16/CD32 (BioLegend, cat#: 101302, clone 93) and incubated for 15 minutes at 4°C. Then 50 µL of D-PBS containing APC-conjugated human PD-L1 mAb (BioLegend cat#: 329708, clone 29E.2A3) and 0.5 µL LIVE/DEAD™ Fixable Yellow Dead Cell Stain (Molecular Probes cat#: L34959) was added. Tubes were then incubated for 30 minutes at 4°C in the dark. After incubation, cells were washed with 2 mL D-PBS via centrifugation at 300 x g for 5 minutes. The supernatant was then poured off and the cells re-suspended and analyzed using a Beckman Coulter Gallios™ flow cytometer. ** p ≤ 0.01; *** p ≤ 0.001 as determined by an unpaired t test (n = 3).

 
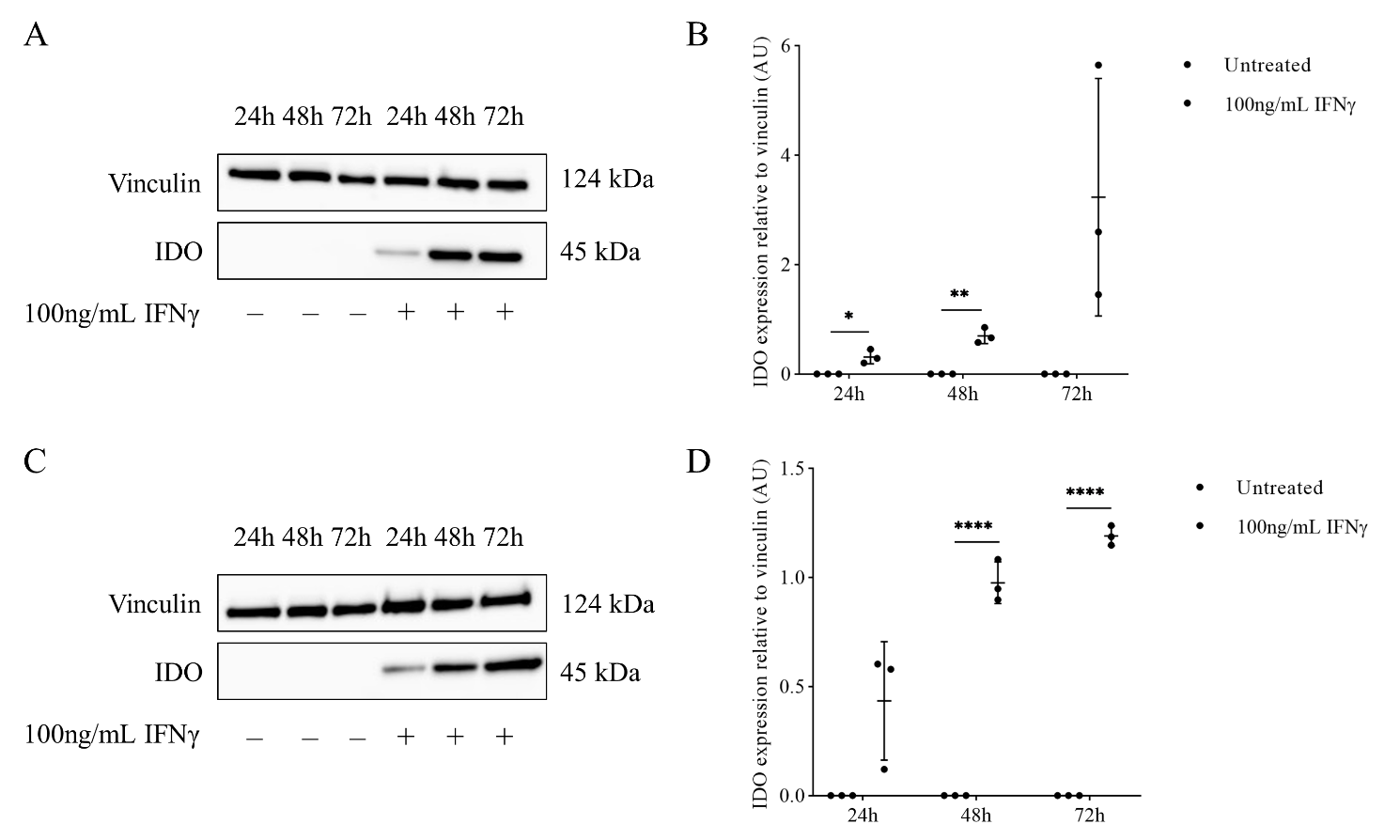


**Supplementary Figure 3.** SEBTA-027 (A and B) and SF-188 (C and D) GBM cell lines actively respond to IFNγ by upregulating IDO. Cells were treated with 100ng/mL of human IFNγ (Peprotech) for 24, 48 and 72 hours and then lysed using radioimmunoprecipitation (RIPA) buffer containing Halt Protease and Phosphatase Inhibitor Cocktail (ThermoFisher). Cells were scraped off the tissue culture flask surface and then transferred to a 1.5 mL tube where they were then vigorously vortexed and placed on ice for 10 minutes – this was repeated two more times. Tubes were centrifuged to separate cellular debris from the lysate and the protein concentration was assessed using a Pierce BCA Protein Assay Kit (ThermoFisher). A 5% stacking gel with 10% resolving gel was made and 30 µg of protein were loaded. Once the samples had been separated via SDS-PAGE proteins were transferred to PVDF membranes, and these were blocked in 5% w/v milk powder in TBS-T for 1 hour at room temperature. Membranes were then incubated in rabbit anti-vinculin mAb (1:10,000, Abcam, cat#: ab129002, clone EPR8185) and mouse anti-IDO mAb (1:250, Abcam, cat#: ab55305, clone 4D2) diluted in 5% w/v milk powder in TBS-T overnight at 4°C. These were then washed 5 times, 5 minutes each time, in TBS-T and then placed in HRP-conjugated goat anti-rabbit (1:1000, Cell Signaling Technology, cat#: 7074) and HRP-conjugated horse anti-mouse (1:1000, Cell Signaling Technology, cat#: 7076) polyclonal antibodies diluted in 5% w/v milk powder in TBS-T for 1 hour at room temperature. Membranes were then washed as previously stated, coated with Clarity Western ECL Substrate (BioRad) and imaged using the Syngene G:Box system (Syngene). ImageJ software was used to analyze the intensity of the bands on the imaged blots and the relative expression was determined by dividing the intensity of the IDO band/the intensity of the vinculin loading control band.
