# Supplementary material for: TRP-2 / gp100 DNA vaccine and PD-1 checkpoint blockade combination for the treatment of intracranial tumors
Source: Cancer Immunol Immunother. 2024 Jul 2;73(9):178. doi: 10.1007/s00262-024-03770-x (PMC11219641; doi:10.1007/s00262-024-03770-x)
Supplement: Supplementary file 2 — Supplementary file2 (PDF 1450 KB) [file 262_2024_3770_MOESM2_ESM.pdf]

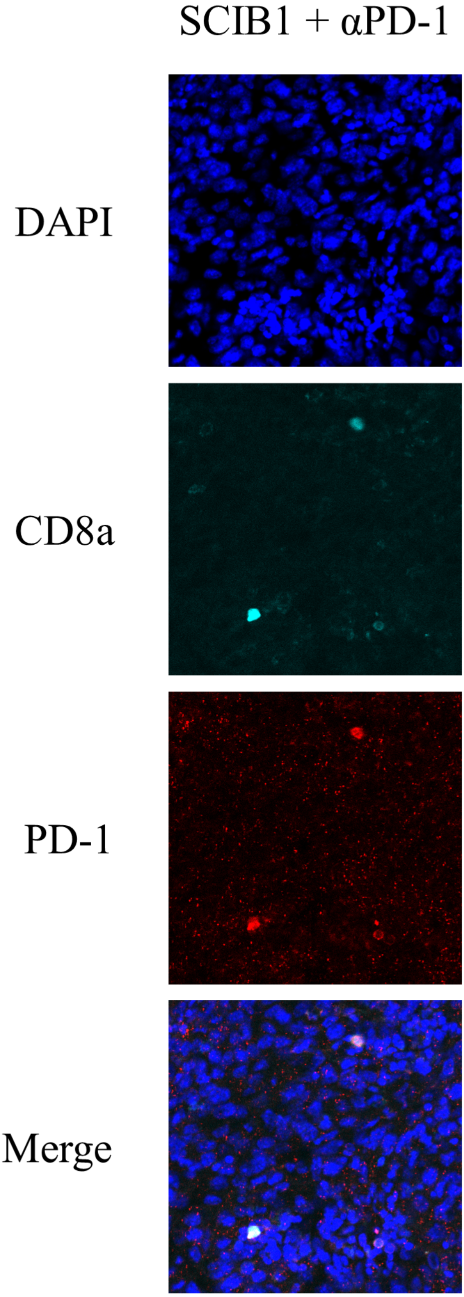

Supplementary Figure 4. PD-1$^+$ CD8$^+$ T cell tumour infiltration in mouse vaccinated with SCIB1 ImmunoBody and anti-PD-1. The cryosectioned brain of a SCIB1 + anti-PD-1 vaccinated mouse was stained for CD8a and PD-1 to investigate T cell exhaustion. The acquired image was processed using ImageJ to quantify CD8$^+$ PD-1$^+$ T cells within the tumour area.
